# The effect of climate variability in the efficacy of the entomopathogenic fungus *Metarhizium acridum* against the desert locust *Schistocerca gregaria*

**DOI:** 10.1038/s41598-022-11424-0

**Published:** 2022-05-09

**Authors:** Samuel F. Kamga, Frank T. Ndjomatchoua, Ritter A. Guimapi, Ingeborg Klingen, Clément Tchawoua, Anne-Grete Roer Hjelkrem, Karl H. Thunes, Francois M. Kakmeni

**Affiliations:** 1 Department of Physics, Faculty of Science, University of Yaoundé 1, P.O. Box 812, Ngoa Ekelle, Yaoundé, Cameroon; 2 Department of Physics, Faculty of Science, University of Buea, P. O. Box 63, Buea, Cameroon; 3 Sustainable Impact Through Rice-Based Systems, International Rice Research Institute (IRRI), DAPO Box 7777-1301, Metro Manila, Philippines; 4 Biotechnology and Plant Health Division, Norwegian Institute of Bioeconomy Research (NIBIO), P.O. Box 115, 1433 Ås, Norway; 5 Division of Food Production and Society, Norwegian Institute of Bioeconomy Research (NIBIO), P.O. Box 115, 1433 Ås, Norway; 6 International Centre of Insect Physiology and Ecology (icipe), P.O. Box 30772-00100, Nairobi, Kenya

**Keywords:** Biogeography, Ecological modelling, Invasive species, Population dynamics, Fungi

## Abstract

Despite substantial efforts to control locusts they remain periodically a major burden in Africa, causing severe yield loss and hence loss of food and income. Distribution maps indicating the value of the basic reproduction number *R*_0_ was used to identify areas where an insect pest can be controlled by a natural enemy. A dynamic process-based mathematical model integrating essential features of a natural enemy and its interaction with the pest is used to generate *R*_0_ risk maps for insect pest outbreaks, using desert locust and the entomopathogenic fungus *Metarhizium acridum* (Synn. *Metarhizium anisoliae var. acridum*) as a case study. This approach provides a tool for evaluating the impact of climatic variables such as temperature and relative humidity and mapping spatial variability on the efficacy of *M. acridum* as a biocontrol agent against desert locust invasion in Africa. Applications of *M. acridum* against desert locust in a few selected African countries including Morocco, Kenya, Mali, and Mauritania through monthly spatial projection of *R*_0_ maps for the prevailing climatic condition are illustrated. By combining mathematical modeling with a geographic information system in a spatiotemporal projection as we do in this study, the field implementation of microbial control against locust in an integrated pest management system may be improved. Finally, the practical utility of this model provides insights that may improve the timing of pesticide application in a selected area where efficacy is highly expected.

## Introduction

For centuries, humans have attempted to control pest insect populations that cause agricultural productions losses, and often, chemical insecticides are used to control these insect pests^1^. Unfortunately, extensive use of chemical pesticides contributes to a plethora of issues such as farmers’ health risks, food safety issues, reduced biodiversity, reduction or loss of natural enemies, pollinators, and other non-target organisms, and emergence of pesticide resistance^2^. Historically, the use of chemical pesticides to control pests made it possible to increase yields^3^ and chemical pesticides will probably continue to be a vital tool that can maintain and improve yields in future sustainable plant production systems^4^ but only in combination with new technologies and non-chemical alternatives^2^. Driven by the desire to develop alternative methods, a number of studies have explored the use of a natural enemies to control insect pests^5,6^. Different biological control methods, as defined by^7^, is considered as an important component of integrated pest management (IPM) in many countries and is mentioned as principle four (non-chemical methods to be preferred) out of the eight principles of IPM in the European Union’s Directive on Sustainable Use of Pesticides (2009/128/EC SUD). Biological control can be defined as the usage of living organisms (predators, parasitoids and entomopathogens) to reduce the density of pest insects^7^. Insect pathogenic fungi are now widely used as biocontrol agents of pests insect in many countries^8–12^.

Microbial control can be viewed as applied epizootiology with the goal of inducing an epizootic incidence, i.e. an outbreak of a disease in which there is an unusually large number of cases in the targeted insect population, through manipulation^13^. Processed-based models development is an ideal tool toward mimicking and predicting the success of a biological process and have been developed to answer biological questions about: (i) predicting the outbreak and success of a specific introduction of a pathogen, (ii) predicting the impact of an introduced pathogen on ecosystems and non-target species, (iii) predicting optimum release and management strategies, (iv) assistance in the selection of the most appropriate agent(s) and, (v) model development that allows a good understanding of the processes involved and the reasons for success or failure^14^. Biocontrol success or failure can be modeled by a set of differential equations with the aim to understand or predict the underlying mechanisms of interaction^14,15^. Challenges of modeling the epizootiological development of an entomopathogenic fungus in an insect population resides on all four primary areas that are known to influence epizootiology: (i) the pathogen population, (ii) the host population, (iii) transmission and, (iv) the environment (biotic and abiotic)^16,17^. Both virulence and pathogenicity are important expressions when modelling and discussing the epizootiological development of an entomopathogenic fungus. We will therfore use the definitions by Lacey^18^ when using these expressions. They go as follows: “Virulence is the speed by which a microorganism penetrates hosts defenses” and “Pathogenicity is the intrinsic capability of a microorganism to penetrate the host defenses”.

*M. anisopliae* var. *acridum* have shown considerable potential when applied as a biocontrol agent of locusts^20–21^ and is also effective against locusts that have developed resistance to chemical insecticides^22^. *M. anisopliae* biopesticide is reported to kill 70%–90% of treated locusts within 14–20 days in field condition^19^. Green Muscle is a biopesticide developed and commercialized since 2001 by LUBILOSA (Lutte Biologique ,contre les Locustes et Sauteriaux) that kill locust and grasshoppers and pose a low risk to the environment^23^ It was successfully used to contain the locust infestation in the Iku-Katavi National Park in Tanzania in 2009; where around 10 000 hectares were treated to protect and ensure the safety of animals (elephants, giraffes and hippopotamuses) within the area^24^. The product was also used during the recent locust outbreak in 2019/2020 to treat more than 230 000 thousand of hectares in the horn of Africa, therefore helping about 20 million people in Kenya, Uganda, South Sudan, Somalia, Ethiopia and Tanzania to fight food insecurity^25^. Although *M. anisopliae* may infect insects living in a wide array of habitats, its performance as a biocontrol agent is highly variable depending on the environmental conditions to which it is exposed^22,26^. Temperature and relative humidity is known to affect the performance of *M anisopliae* significantly^28–30^ and can substantially impact the success of biological control^31^. For instance, it has been demonstrated that temperature significantly affects the fungal germination rate^33–34^, the fungal growth rate within the host^32–34^, sporulation and virulence of this entomopathogenic fungus^35^. The optimal temperature for *M. anisopliae* is concidered to range from 25 to 35 °C^36^.

However, the optimal temperature may vary depending on the geographic, and hence, climatic origin of the isolate and to be able to build good descriptive models for a pest natural enemy system, it is imperative to include information about the temperature in the region where the isolate comes from^37^. Non-optimal temperatures may affect the rate of locust pest mortality by inhibiting spore germination if the temperature is higher than 35 °C or lower than 20 °C*,* which in turn affects penetration through the insect’s cuticle^34^. Relative humidity also affects fungal control agents^27,38^ since it is essential for fungal germination and sporulation on insect cadavers^19,39,40^. The optimal relative humidity levels are suggested to be more similar for isolates of all geographical origins^37^, and most entomopathogenic fungi require about 95% relative humidity at the host surface (microclimate) to germinate^41^. The relative humidity at the host surface where the entomopathogenic fungal conidia germinate may be higher than the ambient relative humidity^37^. Further, tomato greenhouse studies with fungi in the Hypocreales support that a higher humidity is found in the leaf boundary layer (microclimate) and that this benefit microbial control of small arthropod pests living in that stratum^42,43^. Therefore, the effect of microclimatic relative humidity on host surface or in leaf boundary layer should be taken into consideration when using ambient relative humidity from weather stations in modelling, forecasting and decision support systems for when to successfully apply a fungal based biocontrol agent^44,45^. Despite advancing new insights into the importance of climatic variables on the efficiency of entomopathogenic fungi as biocontrol agents^44,45^, few studies have used both temperature and relative humidity as paramount climatic factors to model and predict the ideal conditions for successful use and application of entomopathogenic fungi in the Hypocreales^47–48^.

A key to use entomopathogenic fungi efficiently as a biocontrol agent is to predict how it will perform across space and time. A model used to study the density dependence and spatial structure in the dynamics of an entomopathogenic fungus is presented in^49^ and a description of the behavior of infected and non-infected hosts and the prediction of the relevant spatial scale during the spread of entomopathogenic fungus is presented in^50^. The analysis of the spread of a contagious disease caused by an entomopathogenic fungus in an insect pest population at different host densities is reported in^51^. The understanding of the effect of conidial dispersal of an entomopathogenic fungus on survival of its host is clarified in^52^. The outcomes of these models often give potential geographical areas where an entomopathogenic fungus may perform well but without integrating climatic factors such as temperature and relative humidity. In previous studies, meteorological station data in a Geographical Information System (GIS) were used to investigate the most environmentally suitable condition for a good performance of entomopathogenic fungi over a wide spatial and temporal scale^31^. In^53^ the authors explored climate-driven (moisture, radiation, precipitation and temperature) geographic distribution of the desert locusts during the locust recession period in Africa.

The basic reproduction number, *R*_0_, is a key epidemiological metric for understanding pest risks^54^ by determining the threshold values at which, the model exhibit changes in its stability. For our entomopathogenic fungus system, *R*_0_ is defined as the expected number of new infected individuals (locusts) that a single fungal infected locust may generate in a population of entirely susceptible locusts. Typically, when *R*_0_ is greater than one, the introduced fungus can proliferate among the locust pest population and presumably infect and kill more hosts with time, whereas when *R*_0_ is smaller than one, the locust outbreak effects cannot be attenuated if the fungus is introduced, and the fungus will certainly die out with time. Therefore, the basic reproduction number also indicates the amount of control needed to eradicate locusts during an outbreak. In previous studies, *R*_0_ was used to investigate how the timing and intensity of a virulent entomopathogenic fungus affect insect reproduction and mortality^56–57^. It can provide an approximation of the level of the efficacy of the entomopathogenic fungus towards the pest insect and therefore it offers a good measure for the biocontrol potential of the entomopathogenic fungus towards locusts^58^.

Our research hypothesizes that combining mechanistic model development and experimental biocontrol results using entomopathogenic fungus may improve the strategy and guidelines for effective insect pest control. As a complement to experimental studies which focused on the growth and development of entomopathogenic fungi^20,22,29,31,59,60^, the current study proposes a generic modelling approach to map the dynamics of interactions of the entomopathogenic fungus *Metarhizium acridium* (Synn. *Metarhizium ansiopliae var. acridum)* (Hypocreales) against the desert locusts *Schistocerca gregaria*; considering the effects of climatic factors (temperature and relative humidity) and spatial variability (land cover) when estimating *R*_0_**.** The spatial variation of the pathogenicity is herein explored.

## Results

### Sensitivity analysis

Table 1 displays the Partial Rank Correlation Coefficient (PRCC) for each parameter included in the sensitivity analysis. Four parameters (*e*, *ω*,* α*,* γ*) will be key contributors to the uncertainty of the efficacy and successful application of the entomopathogenic fungus *Metarhizium acridium* against the desert locust *Schistocerca gregaria*. *R*_0_ was highly sensitive to the fungal growth rate and to the proportion of resources allocated (host insect body) towards the colony-forming unit (CFU) production (absolute PRCC > 0.7). This means the efficacy of the biocontrol strategy would be highly influenced by the CFU (mycelia or conidia) concentration in the host insect population. Furthermore, *R*_0_ was moderately sensitive to the conversion rate of resources into CFU and to the mycelium death rate (absolute PRCC > 0.5). Finally, *R*_0_ was less sensitive to the insect carrying capacity (maximum density of pest insect the habitat can support) and the Half-saturation constant (density host insect body at which half of the maximum intake is reached) (absolute PRCC < 0.5).

**Table 1:** PRCC (partial rank correlation coefficient) values and *p* values for each parameter in the *R*_0_ sensitivity analysis.

Parameter description	PRCC	*p* value
Proportion of resources (host insect body) allocated towards spore production (*e*)	(*) + 0.774	0.4715
Fungal growth rate (*ω*)	(*) + 0.764	0.4032
CFU cell background death rate (*α*)	(*) + 0.648	(**) 0.001
Conversion rate of resources (host insect body) into CFU (*γ*)	(*) − 0.634	0.8260
Insect carrying capacity (*K*)	− 0.383	0.8777
Half-saturation constant (*β*)	+ 0.197	0.9345

It summarizes the results and statistical significance in terms of PRCC and *p* value when changing model parameter values. The sign of PRCC represents the positive (+) or negative (−) response of *R*_0_ to the changed parameter values.

(*) denotes PRCCs that are highly different from 0 (− 0.5 ≥ *PRCC* ≥ 0.5).

### Behavior of the basic reproduction number (*R*_0_) with changes on climate variables

**Figure 1:**
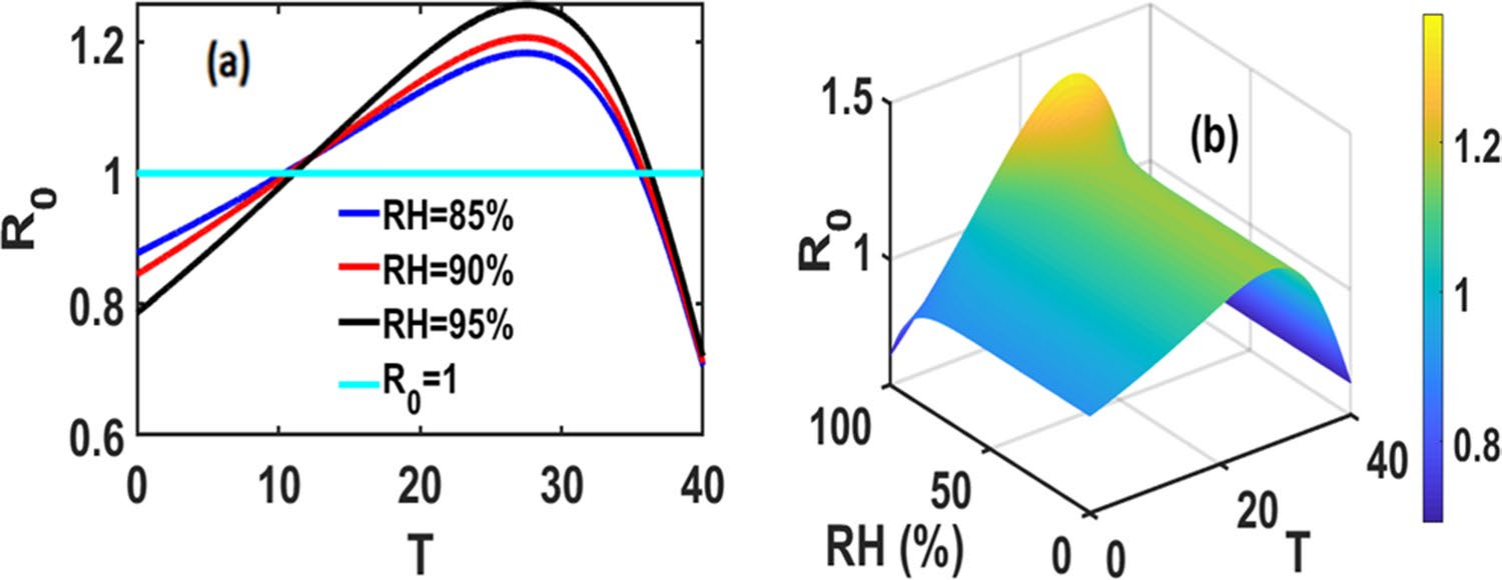
Combined effect of Temperature (T) and Relative Humidity (RH) on basic reproduction number (*R*_0_) expression. (**a**) Dependence of *R*_0_ on temperature at constant RH and (**b**) 3D plot of *R*_0_ as a function of both RH and temperature.

Figure 1a displays the results of the numerical simulation of the dependence of *R*_0_ on temperature with relative humidity fixed at 85, 90 and 95%. The dependence of *R*_0_ on both temperature and relative humidity is plotted in Fig. 1b. Figure 1 suggests that relative humidity only affects the magnitude of *R*_0_ and has no effect on the optimal temperature for efficacy of the entomopathogenic fungus (*M. acridum*). Using the parameters estimated for desert locust (Tables 2, 3), the simulations indicate that the maximum value of *R*_0_ is 1.2 and it is obtained for temperature within the range 24–33 °C at relative humidity above 95%.

The areas where *R*_0_ was close to 1.2 correspond to the area where the *M. acridum* can provide optimal control. In areas where *R*_0_ < 1, desert locust outbreak cannot be attenuated using the entomopathogenic fungus only, whereas the areas were *R*_0_ > 1 M*. acridum* can spread widely and hence be used against an outbreak. Temperatures higher than 35 °C reduce the efficacy of *M. acridum*, the entomopathogenic fungal species in question. Temperature within the range 15–33 °C, *R*_0_ > 1 (Fig. 1a), is suitable for *M. acridum* but is optimal within the range 24–33. The maximum *R*_0_ value of is reached at 28 °C and 95% or higher relative humidity (Fig. 1a,b), which also corresponds to the value in which the fungal development rate and the proportion of resource allocated for spore production are optimal.

### Spatiotemporal projection in distribution the basic reproduction number (*R*_0_)

The spatial projection and the mapping of the variation in *R*_0_ reflecting the efficiency of the entomopathogenic fungus *M. acridum* as a biocontrol agent across selected African countries are shown in the maps representing distribution inferred by the degree of magnitude of *R*_0_ that indicates higher values in most areas where locusts outbreak can be attenuated after introduction of *M. acridum* (*R*_0_ > 1).

**Figure 2:**
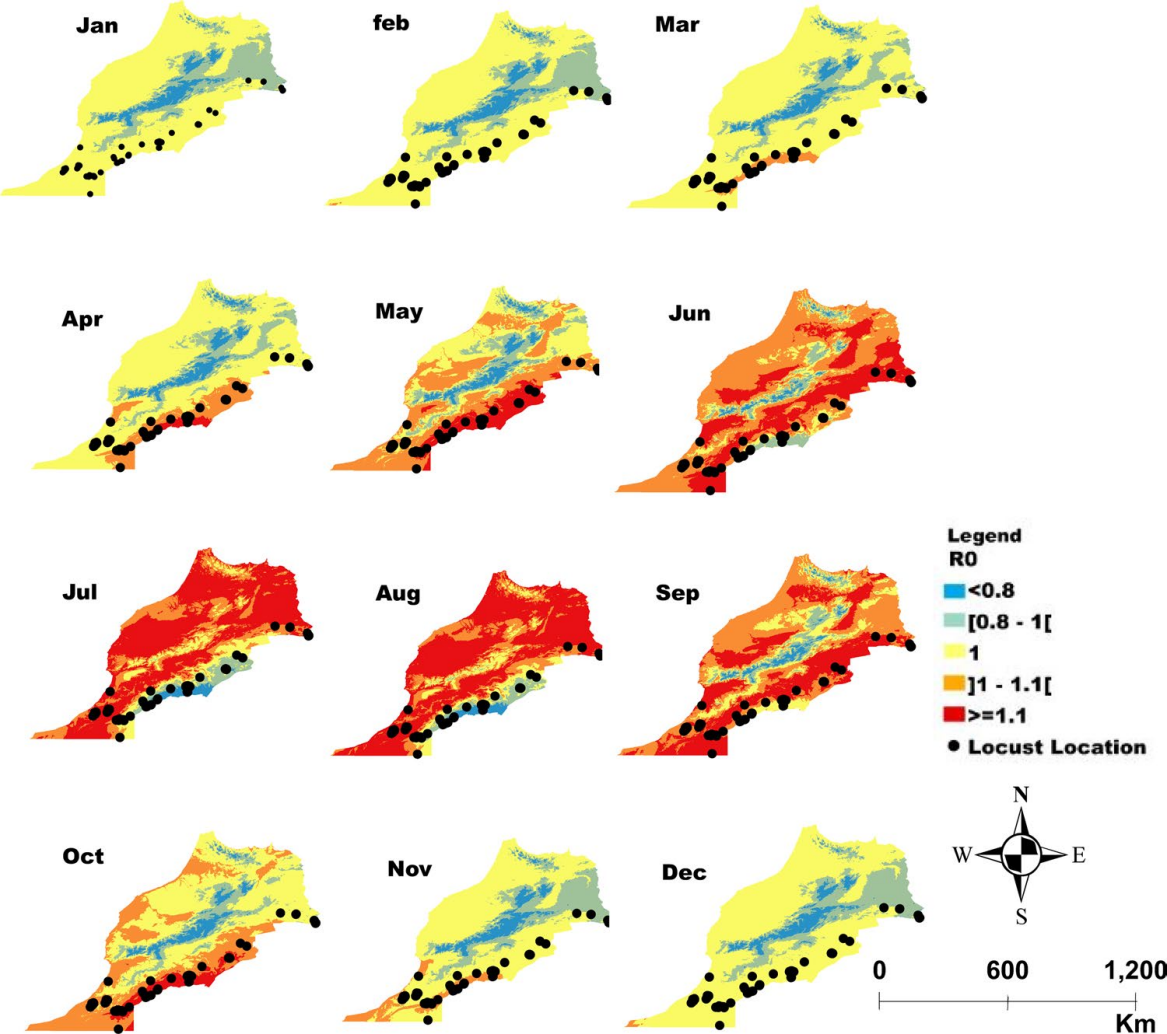
Morocco: Monthly spatial projection of the variation in *R*_0_ as a function of temperature and relative humidity reflecting the potential efficiency of the entomopathogenic fungus *Metarhizium acridum* as a biocontrol agent against desert locust. The area in red in each month are areas where *R*_0_ > 1 correspond to zones with potential higher efficiency and hence successful fungal biocontrol agent application against locusts while the area in orange (*R*_0_* ≈* 1) can also be targeted but with less potential efficacy. Application in areas in yellow and blue (*R*_0_ < 1) will probably not be efficient. The dots correspond to the reported point incidence of locusts in Morocco.

**Figure 3:**
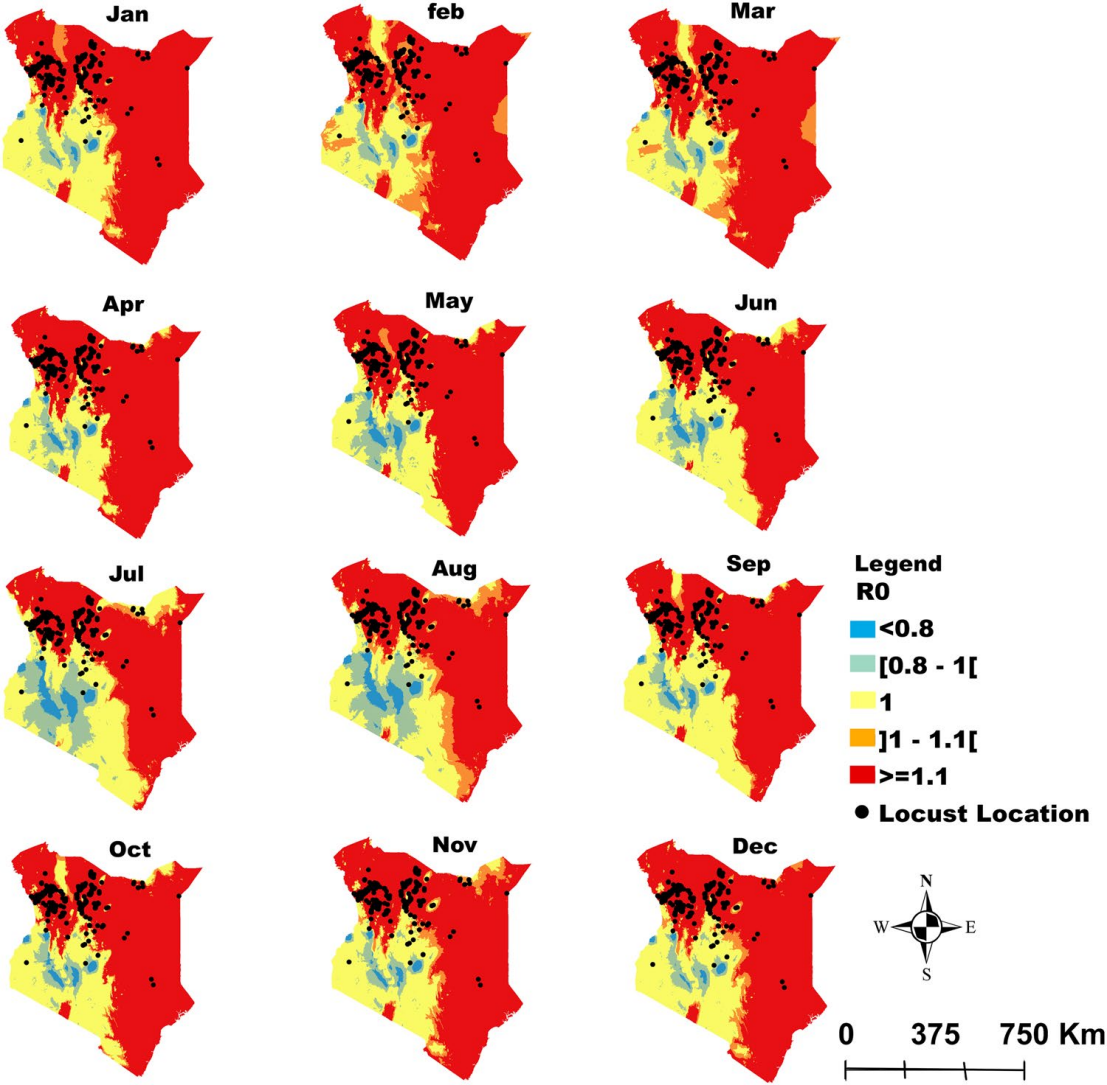
Kenya: Monthly spatial projection of the variation in *R*_0_ as a function of temperature and relative humidity reflecting the potential efficiency of the entomopathogenic fungus *Metarhizium acridum* as a biocontrol agent against locusts. The area in red in each month are areas where *R*_0_ > 1 correspond to zones with potential higher efficiency and hence fungal biocontrol agent application against desert locusts while area in orange (*R*_0_ *≈* 1) can also be targeted but with less potential efficacy. Application in areas in yellow and blue (*R*_0_ < 1) will probably not be efficient. The dots correspond to the reported point incidence of locusts in Kenya.

**Figure 4:**
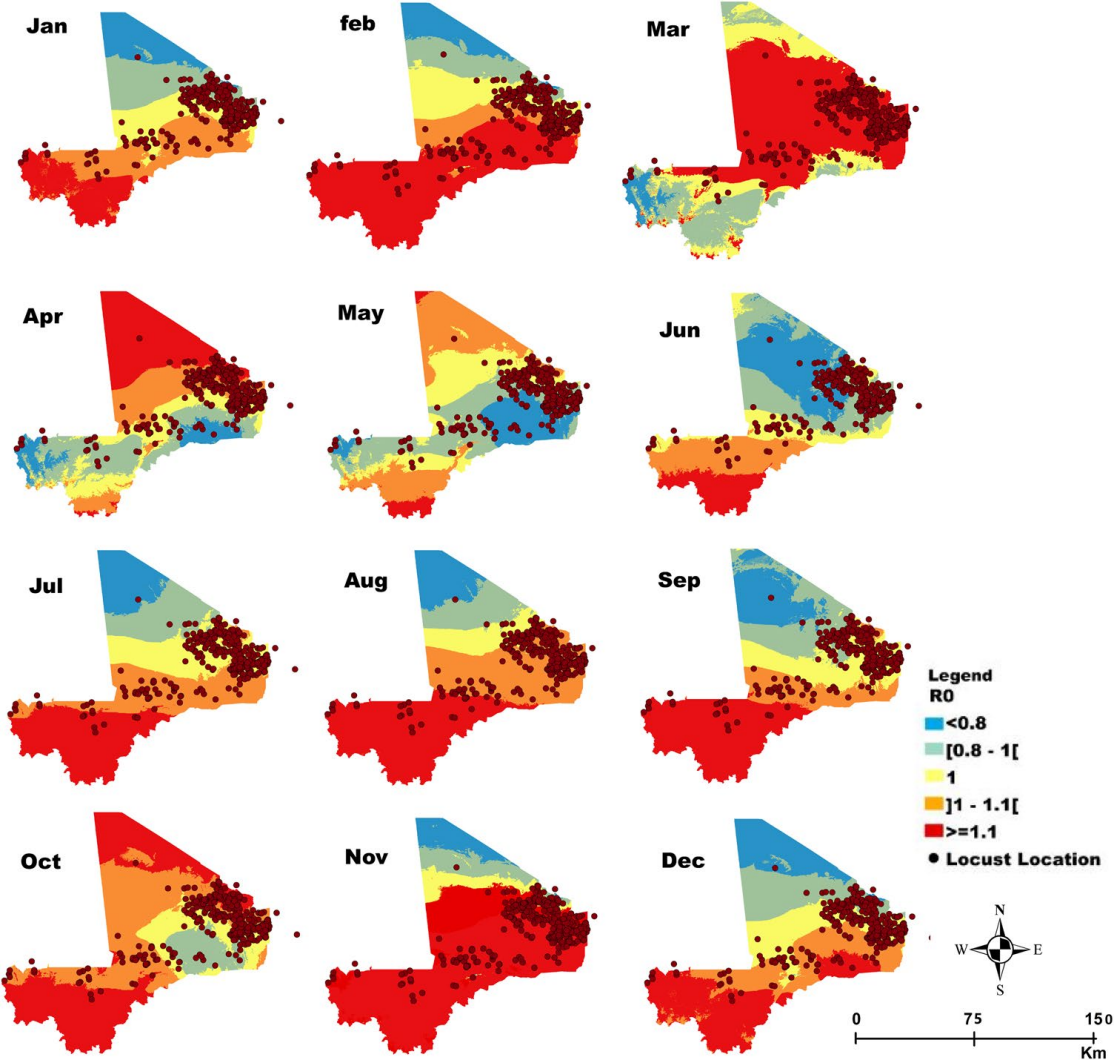
Mali: Monthly spatial projection of the variation in *R*_0_ as a function of temperature and relative humidity reflecting the potential efficiency of the entomopathogenic fungus *Metarhizium acridum* as a biocontrol agent against locusts. The area in red in each month are areas where *R*_0_ > 1 correspond to zones with potential higher efficiency and hence fungal biocontrol agent application against locusts while the area in brown (*R*_0_ *≈* 1) can also been targeted but with less potential efficacy. Application in areas in yellow and blue (*R*_0_ < 1) will probably not be efficient. The dots correspond to the reported point incidence of locusts in Mali.

**Figure 5:**
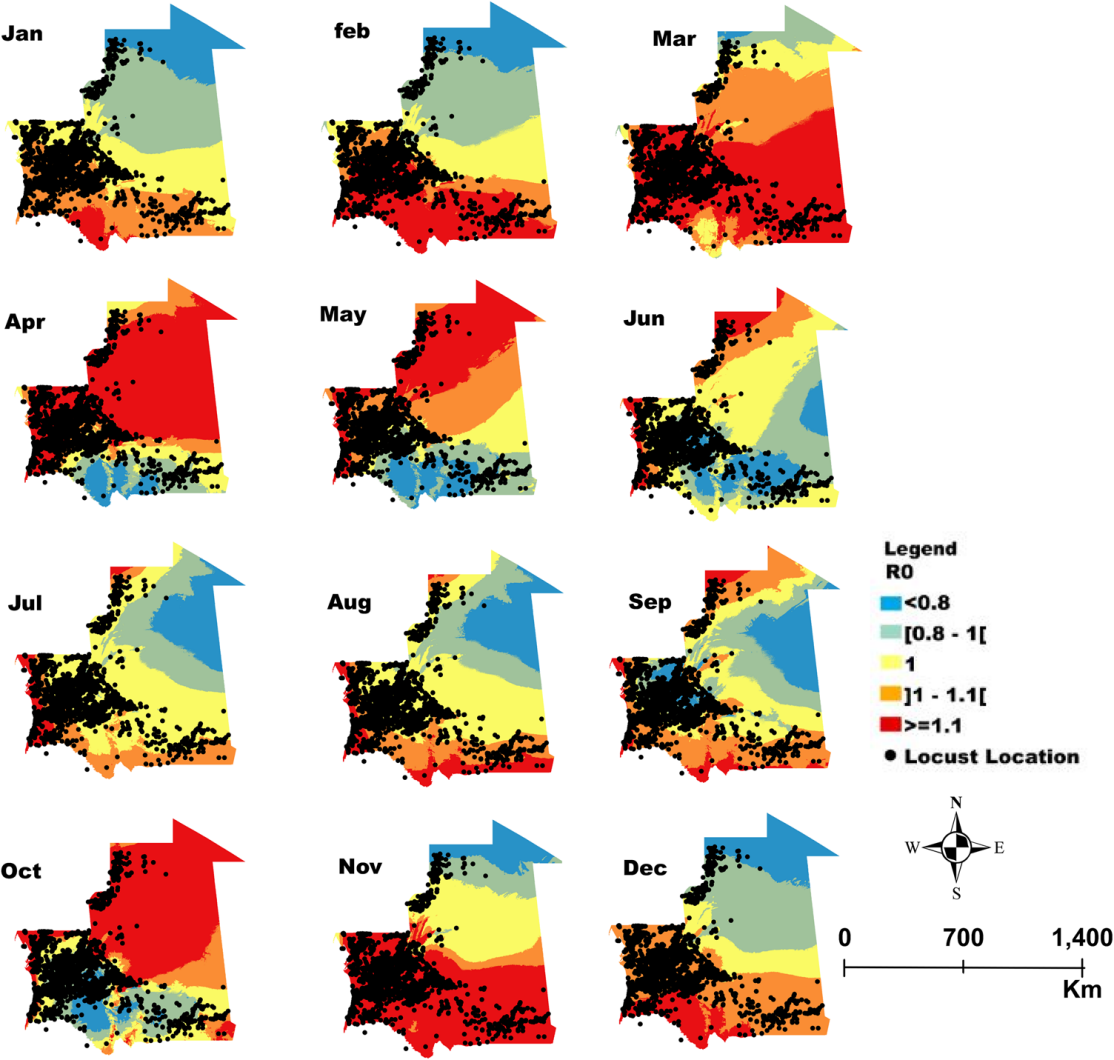
Mauritania: Monthly spatial projection of the variation in *R*_0_ as a function of temperature and relative humidity reflecting the potential efficiency of the entomopathogenic fungus *Metarhizium acridum* as a biocontrol agent against desert locusts. The area in red in each month are areas where *R*_0_ > 1 correspond to zones with potential higher efficiency and hence fungal biocontrol agent application against locusts while area in orange (*R*_0_ *≈* 1) can also be targeted but with less potential efficacy. Application in areas in yellow and blue (*R*_0_ < 1) will probably not be efficient. The dots correspond to the reported point incidence of locusts in Mauritania.

Figures 2, 3, 4 and 5 display the monthly variation of *R*_0_ across, Morocco, Kenya, Mali and Mauritania respectively. The locations in red colors (values of *R*_0_ > 1) represent the areas that may be suggested for application of *M. acridum* against desert locust outbreaks that month. Suggested locations vary from one month to another following climate variability within the respective countries and correspond to areas where *M. acridum* spore production would multiply exponentially and be transmitted for the period the environmental conditions are favorable.

## Discussion

Developing mathematical models for understanding the long term dynamic of climatic variables affecting the efficacy and successful application of an entomopathogenic fungus as a control agent of desert locust is a task that necessitates a robust conceptual framework, capable of exploring population dynamics both temporally and spatially. The present study proposed a modelling framework to help in exploring the spatial variability in performance of *M. acridum* applied as a biocontrol agent of desert locusts and apply it in selected African countries where locust outbreaks are common. Our model study confirmed that relative humidity and temperature are key factors impacting the biocontrol potential for the entomopathogenic fungus *M. acridum* to infect and kill the desert locust.

Processed-based modeling has been used to describe the mechanisms responsible for the dynamics of factors affecting the epidemiology of a entomopathogenic fungus^14,61^. The effect of time on fungal-pest interactions has been modeled by various forms of differential equations considering discrete time maps^62^, metapopulations^63^, networks and spatial data^14^. Developing reliable modeling frameworks which account for the prediction of large-scale climatic variables such as temperature and relative humidity on the performance of entomopathogenic fungi can be useful to schedule guidelines for smallholder farmer in the implementation of suitable pest management strategies.

In previous studies, a simulation model that captures the effects of temperature and host-mediated behavior was presented^31^, the effect of relative humidity on the performance of fungus for controlling locusts and grasshoppers using linear Eqs. ^38^ was explored. Through these models, the authors showed that high temperature (above 35 °C) is a limiting factor for the performance of the entomopathogenic fungal isolates they tested; Klass et al.^31^ on *M. anisopliae* (Hypocreales) and Hajek et al.^38^ *Entomophaga maimaga* (Entomophthoromycota), but that high relative humidity was required for the isolates to perform well. These models are based on the association between environmental factors (temperature) and the spatial arrangement of the fungus, aiming to identify the conditions most suitable for the species (*M. anisopliae* or? *E. maimaga*) to control locusts. The favorable conditions identified by these models were projected into geographical space to infer spatial assumptions (based on temperature) about the possible distribution of the entomopathogenic fungi, to generate maps that shows the variation of the lethal time towards the host (locusts) over wider spatial and temporal scales^36^. The derivation and the mapping of an efficient pathogenicity index from a population model unifying essential features of the locust-natural enemy interactions mechanism and integrating jointly the relative humidity and temperature require more investigation.

Controlling locust invasions associated with climate variability will be critical in alleviating the potential threats to global food security that may lead to long-term nutritional emergencies and food crisis^64^. Previous studies reported that the optimum temperature for the efficient growth of most entomopathogenic fungi in the Hypocreales was around 30 °C^19,22,31,36,65,66^, and others found out that most entomopathogenic fungi in the Hypocreales require at least 95% relative humidity at the surface of the insect to germinate^41^. Our numerical results suggested that *R*_0_ is at maximum for temperatures in the range of 24–33 °C at relative humidity above 95%, which agree to a certain level with the experimental results obtained in e.g.^19,22,36,41,65,66^. The accuracy of the model lies in the ability to reproduce almost all the areas where the entomopathogenic fungus can spread and then successfully control locust pest.

The main goal of locust control is to successfully implement a preventive and proactive strategy that can disrupt their breeding cycle^19^. Furthermore, according to the locust expert at FAO, Keith Cressman, desert locusts can multiply 20-fold with a new generation every three months. Therefore, it is crucial to develop^67^ an effective tool that can help farmers and governments in their field monitoring activities, by building models that may contribute to future decision support systems (DSS) for when and where to apply *M. acridum* to control locusts. Although field validation of the model was not conducted, we aim to achieve this in future collaborations with governments and organizations such as FAO through the team in charge of predicting the migratory pattern of desert locusts across Africa through an early warning system. Once a future locust invasion is forecast in an uninvaded country, an early or preventive application of the biopesticide can be scheduled in a timely manner for areas predicted by this model to be more suitable for surivival of the fungus. To obtain validation data for our model, this should be accompanied with recording field data on locust mortality caused by *M. acridum* and the actual environmental factors before, during and after *M. acridum* application.This should be compared with data from areas without application of *M. acridum* to rule out the effect of naturally occurring *M. acridum* infections of locusts. A preliminary validation can also be performed before investing a lot of resources into real field trials by using the current model to generate the map of Somalia and compare predicted potential efficiency of the area that fits with the 236 000 hectares treated by FAO during the recent locust outbreak^67^.

In Kimathi et al.^68^, the developed ecological niche model spatially projected to Kenya identified five classes (very low, low, moderate, high, very high) of suitability for the locust breeding site classified as [0–0.2] (very low) to [0.8–1.0] (very high). Analyzing the map of efficacy obtained for Kenya in this study and comparing the areas predicted by our model with potential higher efficiency for fungal application against locusts, we realize that there is a match between these areas and the ones with very high suitability for locust breeding^68^. This includes counties such as Garissa, Wajir, Marsabit and Mandera where locust invasions were recently reported. The present study therefore complements the work of Kimathi et al.^68^ by using the variability of the *R*_0_ value to guide where and when it might be advisable to plan the application of the microbial control agent *M. acridum* against desert locust at a large spatial scale. The decrease of *R*_0_ that spatially characterizes the areas with lower efficacy (*R*_0_ < 1) also matches with locations predicted by the ecological niche model as with low or very low suitability for locust breeding and should not be given priority. However, considering the fact that the current spatial projections use climate data collected and interpolated from weather stations, the areas predicted to have moderate efficacy (*R*_0_ *≈* 1) by our model might provide microclimatic conditions adequate for the efficacy of the fungal pathogen against desert locust as suggested by previous studies^37,42^.

In previous studies, several authors have used different approaches to tackle pest insects problems similar to the current study^64,70–78^. For instance, Moukam Kakmeni et al.^72^ used the same method as in this work to derive the spatial distribution maps for malaria transmission under different climatic and intervention scenarios. However, their context required *R*_0_ < *1* for efficient control of the vector-borne diseases which is the opposite to this study where the target is *R*_0_ > *1*. In Klass et al.^31^, the spatial variation of pathogen pathogenicity and the implications for biocontrol of locusts and grasshoppers was explored. Although the outcome is realistic, their method did not include the dynamics of fungus-pest insect interactions and neither considered the effect of relative humidity nor the efficacy of the entomopathogenic fungus. Our study is innovative by its way of using the basic reproduction number *R*_0_ derived from a dynamical mathematical model depending on both temperature and relative humidity to study the efficacy of an entomopathogenic fungus against pest locusts. The study in^56–57^ used a similar approach but the limitation of their studies are that they did not consider the effects of climatic variables, limiting their predictive accuracy.

Our results underline that, realistic models need to include a broad spectrum of spatial and temporal factors to predict the efficacy of a fungal based biocontrol agent with high accuracy. A key challenge is to choose the most appropriate scale for modeling. This is influenced not only by the resolution of available climate data, but also by the biological knowledge that modeling process may translate into something globally observable at varying scale. Note that the greatest effect of climate change on the efficacy of a fungal based biocontrol agent is likely to be observed at the extremes of the optimal range of temperatures and RH at which the maximal fungal growth and germination occurs, which in our system is set at 28 °C and RH above 95%.

By applying the basic reproduction number *R*_0_, maps that illustrate the possibility of a successful application of the fungal based biocontrol agent *M. acridum* for the control of desert locust were obtained. The outcome of this study could constitute a realistic basis for understanding the interactions and complexities between the entomopathogenic fungus and desert locust. By developing models that may increase the efficacy of fungal based biocontrol agents, this may provide farmers with a better IPM tool and hence reduce the use of chemical insecticides as well as concerns connected to their side effects on health and environment.

There are increasing commercialization and farmer adoption of Low Risk Plant Protection Product (Annex II, point 5 of Regulation (EC) 1107/2009) to control locust outbreaks in Africa^67^. The fungal based product Green Muscle (with the active ingredient (a.i.) *M. acridum)* is the most widely used^79^ and reported to be very effective in killing adults within one to two weeks depending on the environmental conditions^67^. Large-scale applications in targeted countries are usually done with planes using oil-based formulation of the product which offer better chance of contact with the locust and makes it less vulnerable to low relative humidity. Since the timing of the spraying of these products is a key for better efficacy, the maps projecting the potential area of efficacy like those presented as output of this model can be useful resources when planning for such activities. This might also help optimizing the allocated resources and focus on specific areas in a specific month where high efficacy is expected.

Looking forward, an improvement from the current state of this work would consider the effect of another environmental factor such as ultraviolet radiation and, also extend similar model development exercises using other entomopathogenic fungi. Moreover, downscaling the spatial extent of the predictions combined with adopting hourly and daily temporal variation would also help improve the accuracy and help agricultural officers to provide reliable advises to farmers.

## Materials and methods

### Study area and datasets used

The areas chosen for the study include Morocco, Mauritania, Kenya and Mali where both past and recent desert locusts outbreaks occurred and were documented with high levels of crop damage and represented as reported point coordinates of desert locust incidences^53,68,80,81^. The datasets used include the average monthly temperatures, the average monthly relative humidity, and the reported point coordinates of desert locust incidence in the respective countries of interest. Temperature data were taken from the Climatologies at High resolution for the Earth Land Surface Areas (CHELSA) climate data (Version 1.2)^82^; corresponding to the monthly average mean for the time period 1979–2013. Relative humidity data were obtained from the NASA Surface meteorology and Solar Energy (SSE) (http://eosweb.larc.nasa.gov/sse/) and corresponded to 22 years mean average. Temperature and relative humidity data used are in raster format.

#### Assumptions

(i) Temperature and macroclimatic relative humidity were considered as the key factors affecting the efficacy of the entomopathogenic fungus *M. acridium* against desert locust.
(ii) Application of the entomopathogenic fungus is successful in the target desert locus infested area and fungal spores are in contact with the desert locust cuticula.
(iii) Application of the entomopathogenic fungus is done at large spatial scale with mixture of different type of land coverage

### Mathematical model

Equations for modeling the epizootiological development of an insect pathogen in an insect pest population often adopt deterministic or stochastic transmission formulation embedded within static or fluctuating environments^14^. The model presented in Gilchrist et al.^62^ is used as root of our new model formulation. Their model originally assumes that the density of the pest within a patch is fixed and the interaction between fungus and the host insect population (desert locust in this context) varies with the time. They characterized the model by three coupled equations describing the host insect population density within a patch, colony forming unit (CFU) being mycelium or conidial concentration in the host insect population (Table 2). In our proposed model the following assumptions are made: (i) the density of host insect pest within a patch is not limited; (ii) the fungus-insect pest interaction depends on the climate variables relative humidity and temperature which influences the epizootiology development within-patch.

**Figure 6:**
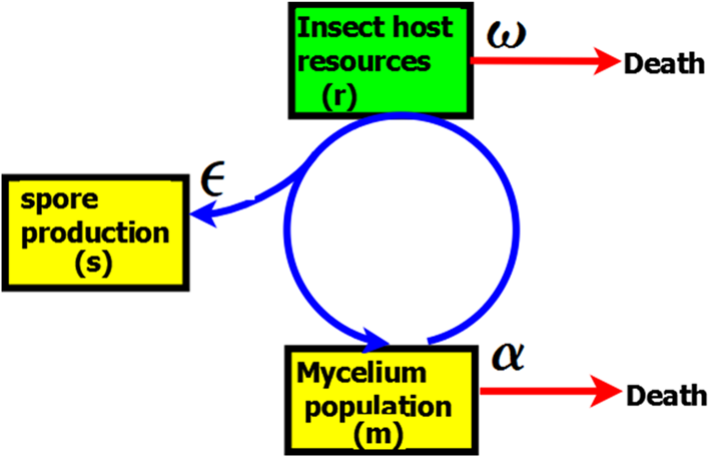
Model diagram of the within-patch system describing the interactions between the entomopathogenic fungus and host insect inside a patch model. Yellow color on the diagram denotes the entomopathogenic fungus M. acridium, green color denotes host insect population (desert locust), blue circle denotes interactions between the entomopathogenic fungus and the host insect, red arrow denotes death. See Table 2 for detailed model explanation and parameters definitions (adapted from^62^).

The interaction between pest insect density (*r*) within a patch and the concentration of CFUs (*m*) (Fig. 6) is described at a daily time resolution (*t*) by the following coupled differential equations:

1 $$\frac{dr}{{dt}} = c_{0} r\left( {1 - \frac{r}{K}} \right) - \left( {1 - \omega } \right)\frac{r}{\beta + r}m$$

2 $$\frac{dm}{{dt}} = m\left( {\gamma \left( {1 - \varepsilon } \right)\left( {1 - \omega } \right)\frac{r}{\beta + r} - \alpha } \right)$$

where *c*_0_, *β*, *K*, *α* and *γ* are fixed model parameters (see Table 2) while ω and ε are estimated based on equations presented in Table 3.

**Table 2:** List and definition of model parameters.

Parameter	Symbol	Value	Source
CFU death rate of entomopathogenic fungus	*α*	0.66	^62^
Half-saturation constant of the density of host insect	*β*	0.32	^57^
Conversion rate of host insect into mycelium of entomopathogenic fungus	*γ*	1.0	^62^
Conversion rate of host insect into conidia of entomopathogenic fungus	*c*_1_	1.0	^62^
Entomopathogenic fungal growth rate	*ω*	Estimated	^31^
Proportion of resources allocated for conidia production	*e*	Estimated	^60^
Insect carrying capacity	*K*	1.0	^57^
Intrinsic growth rate of insect	*c*_0_	1.0	^57^

Moreover, the spore production rate of the fungus *s*(*t*), is proportional to the amount of growth media (pest insect body) available for spore production by the fungus (see Fig. 6)^62^. Thus,

3 $$s\left( t \right) = c_{1} \varepsilon \left( {1 - \omega } \right)\frac{r}{\beta + r}m.$$

See Table 2 for a list of parameters of this within-patch model.

### Equilibrium points and stability analysis

The equilibrium points are found by solving the equations $dr/dt = dm/dt = 0$ of the within-patch models Eqs. (1) and (2). The equation has three steady state points: (i) the trivial steady state, (ii) the pathogen-free equilibrium point and (iii) the basic reproduction number *R*_0_. To support the local stability of the steady states, we examine the linearized form of Eq. (1) at the equilibrium points.

#### The trivial steady state

The trivial steady state of the within-patch models Eqs. (1) and (2) is given by $E_{0} = \left( {r,m} \right) = \left( {0,0} \right)$. *E*_0_ is a saddle point, and at this point, there is no insect pest or entomopathogenic fungus. The Jacobian matrix of the within-patch model Eqs. (1) and (2) at the point is given by:

4 $$J_{{E_{0} }} = \left( {\begin{array}{*{20}c} {c_{0} } & 0 \\ 0 & { - \alpha } \\ \end{array} } \right)$$

with the characteristic equation:

5 $$\lambda^{2} + \left( {\alpha - c_{0} } \right)\lambda - \alpha c_{0} = 0$$

where the eigenvalues (λ) are given by $\lambda_{1} = - \alpha$, $\lambda_{2} = c_{0}$.

#### The pathogen-free equilibrium point

The pathogen-free equilibrium point is given by $E_{1} = \left( {r,m} \right) = \left( {K,0} \right)$. At this point, there are no fungal infected pest insects. The Jacobian matrix of the within-patch models Eqs. (1) and (2) at this point is given by:

6 $$J_{{E_{1} }} = \left( {\begin{array}{*{20}c} { - c_{0} } & {\frac{{\left( {\omega - 1} \right)K}}{\beta + K}} \\ 0 & {\frac{{\gamma K\left( {1 - \varepsilon } \right)\left( {1 - \omega } \right)}}{\beta + K} - \alpha } \\ \end{array} } \right).$$

The characteristic equation corresponding to the pathogen-free equilibrium point *E*_1_ is given by

7 $$\lambda^{2} - \frac{{\left( {K\gamma \left( {1 - \omega } \right)\left( {1 - \varepsilon } \right) - \left( {K + \beta } \right)\left( {\alpha + c_{0} } \right)} \right)}}{\beta + K}\lambda - \frac{{\left( {K\gamma - K\gamma \omega - K\gamma \varepsilon + K\gamma \varepsilon \omega - K\alpha - \alpha \beta } \right)}}{\beta + K}c_{0} = 0.$$

The Routh–Hurwitz criterion ensures that all roots of the polynomial given by Eq. (7) have negative real parts^83^. Using Routh–Hurwitz, the pathogen free-equilibrium point *E*_1_ is stable if the following conditions are satisfied:

8 $$0 < - \frac{{K\gamma - K\gamma \omega - K\gamma \varepsilon + K\gamma \varepsilon \omega - K\alpha - \alpha \beta - c_{0} \beta - c_{0} K}}{\beta + K}$$

9 $$0 < - \frac{{\left( {K\gamma - K\gamma \omega - K\gamma \varepsilon + K\gamma \varepsilon \omega - K\alpha - \alpha \beta } \right)c_{0} }}{\beta + K}.$$

**Figure 7:**
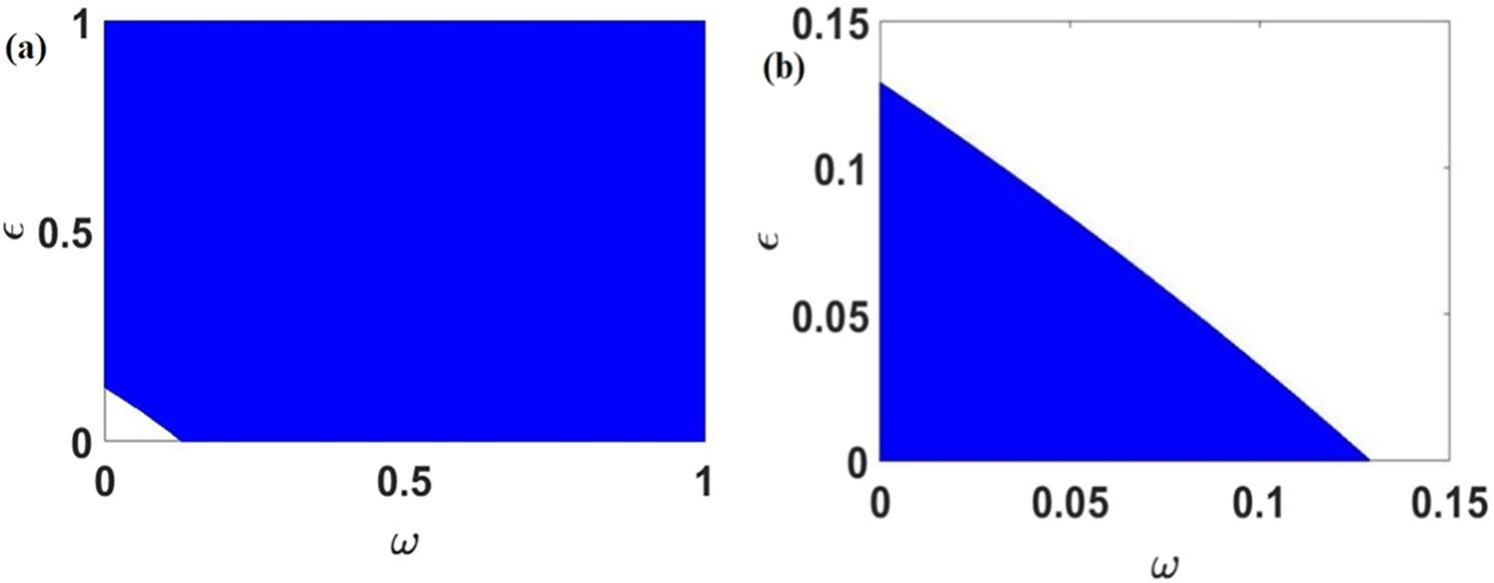
Illustration of the stability diagram of $(\omega ,\varepsilon )$ for within patch model using parameters values in Table 2. (**a**) Stability diagram of the pathogen free equilibrium point and, (**b**) stability diagram of the endemic equilibrium point.

Figure 7a displays the stability diagram of the pathogen free-equilibrium point *E*_1_ given by Eqs. (8) and (9). For the simulations we chose parameters values given in Table 2. The diagram presents a blue region in the parameters space of (*ω*, ε) for, which all roots of the polynomial given by Eq. (7) have negative real parts and therefore corresponding to the stability zone of the pathogen free-equilibrium points.

#### Basic reproduction number *R*_0_

An important metric of interacting and dynamic epizootiology systems is the basic reproduction number *R*_0_. It is applied in this context to quantify how frequently the entomopathogenic fungus transmission occurs and what it will result in (e.g. mortality of insect, reduced reproduction potential).

The basic reproduction number *R*_0_ is further assumed to describe the propensity of the entomopathogenic fungus to survive and to be propagated or go extinct. *R*_0_ is calculated for the proposed model by examining the stability of the pathogen-free equilibrium point $\left( {E_{1} = \left( {r,m} \right) = \left( {K,0} \right)} \right)$. The next generation operator approach described in Hartemink et al.^58^ is employed to evaluate *R*_0_. The formulation of the basic reproduction number *R*_0_ is given by the following expression:

10 $$R_{0} = \frac{{\alpha \left( {\beta + K} \right)}}{{\gamma K\left( {1 - \omega } \right)\left( {1 - \varepsilon } \right)}}.$$

It is shown in Eqs. (8) and (9) that, if *R*_0_ < 1, then the pathogen-free equilibrium is stable and hence the entomopathogenic fungus goes extinct, while if *R*_0_ > 1 then the equilibrium is unstable and hence the entomopathogenic fungus continues to transmit the disease. Estimation of *R*_0_ helps to determine whether locust outbreak effects can be attenuated if the entomopathogenic fungi is introduced (*R*_0_ > 1).

#### The endemic equilibrium point

At this point, both pest insect and entomopathogenic fungus exist. The endemic equilibrium point (*E*_2_) for the within-patch model Eqs. (1) and (2) is given by:

11 $$E_{2} = \left( {\frac{\alpha \beta }{{\gamma \left( {1 - \omega } \right)\left( {1 - \varepsilon } \right) - \alpha }},\frac{{\left( {1 - \varepsilon } \right)\left( {K\gamma \left( {1 - \omega } \right)\left( {1 - \varepsilon } \right) - \alpha \left( {K - \beta } \right)} \right)\gamma c_{0} \beta }}{{K\left( {\gamma \left( {1 - \omega } \right)\left( {1 - \varepsilon } \right) - \alpha } \right)^{2} }}} \right).$$

The Jacobian matrix of the within-patch model Eqs. (1) and (2) at the endemic equilibrium point *E*_2_ is given by:

12 $$J_{{E_{2} }} = \left( {\begin{array}{*{20}c} {j_{11} } & {j_{12} } \\ {j_{21} } & 0 \\ \end{array} } \right)$$

where

13 $$j_{11} = \frac{{c_{0} \alpha \left( {\gamma \left( {1 - \omega } \right)\left( {1 - \varepsilon } \right)\left( {\beta - K} \right) - \alpha \left( {K + \beta } \right)} \right)}}{{\gamma K\left( {\omega - 1} \right)\left( {\varepsilon - 1} \right)\left( {\gamma \left( {1 - \omega } \right)\left( {1 - \varepsilon } \right) - \alpha } \right)}}$$

14 $$j_{12} = \frac{\alpha }{{\left( {\varepsilon - 1} \right)}}\gamma$$

15 $$j_{21} = \frac{{\left( {K\gamma \left( {\omega - 1} \right)\left( {\varepsilon - 1} \right) - \alpha \left( {K + \beta } \right)} \right)c_{0} }}{{\left( {\omega - 1} \right)K}}.$$

The characteristic equation corresponding to the endemic equilibrium point *E*_2_ is expressed as:

16 $$\lambda^{2} - P_{1} \lambda + P_{2} = 0$$

where

17 $$P_{1} = \frac{{c_{0} \alpha \left( {\gamma \left( {1 - \omega } \right)\left( {\varepsilon - 1} \right)\left( {\beta - K} \right) - \alpha \left( {K + \beta } \right)} \right)}}{{\gamma K\left( {\omega - 1} \right)\left( {\varepsilon - 1} \right)\left( {\gamma \left( {1 - \omega } \right)\left( {1 - \varepsilon } \right) - \alpha } \right)}}$$

18 $$P_{2} = \frac{{\left( {K\gamma \left( {1 - \varepsilon } \right)\left( {1 - \omega } \right) - \alpha \left( {K + \beta } \right)} \right)c_{0} \alpha }}{{\gamma K\left( { - 1 + \omega } \right)\left( { - 1 + \varepsilon } \right)}}.$$

Using the Routh–Hurwitz criterion, the endemic equilibrium point *E*_2_ is stable if the following conditions are satisfied:

19 $$0 < \frac{{\left( { - K\gamma \varepsilon \omega + K\gamma \varepsilon + \alpha \beta - K\gamma + K\gamma \omega + K\alpha } \right)c_{0} \alpha }}{{\left( {1 - \omega } \right)\left( {1 - \varepsilon } \right)K\gamma }}$$

20 $$0 < \frac{{c_{0} \alpha \left( { - \beta \gamma \varepsilon \omega + K\gamma \varepsilon \omega + \beta \gamma \varepsilon - K\gamma \varepsilon + \beta \gamma \omega - K\gamma \omega - \gamma \beta + K\gamma - K\alpha - \alpha \beta } \right)}}{{\gamma K\left( {1 - \omega } \right)\left( {1 - \varepsilon } \right)\left( {\gamma - \gamma \omega - \gamma \varepsilon + \gamma \varepsilon \omega - \alpha } \right)}}$$

**Table 3:** Expression of ε and *ω* as a function of temperature and relative humidity.

Variables	Mathematical expression	Estimate of parameter	Source
*ω*	$e^{\rho T} - e^{{\rho k - \frac{{\left( {k - T} \right)}}{{\Delta }}}} + \lambda$	ρ = 0.00938	^31^
		*k* = 266.8810	
		*Δ* = 111.1423	
		λ = − 0.8676	
ε	$10^{ - 4} e^{13.94RH} \left( {e^{\rho T} - e^{{\rho k - \frac{{\left( {k - T} \right)}}{{\Delta }}}} + \lambda } \right)$	ρ = 0.0015	Estimated from data of^60^
		*k* = 266.8810	
		*Δ* = 111.1423	
		λ = − 0.8676	

*ω*(*T*) is the daily fungal growth rate at temperature *T*, ε(*T*,*RH*) is the proportion of fungal growth media (pest insects) allocated towards CFU (mycelium/conidia) (per day) at temperature *T* and *relative humidity*, ρ describes the acceleration rate of the lethal time from the low to optimal temperature; *k* represents the thermal maximum threshold, *Δ* describes the temperature range over which thermal breakdown becomes an over-riding influence; *λ* is the asymptote to which the function tends at the thermal minimum threshold^31^.

Figure 7b illustrates the stability diagram of the endemic equilibrium point *E*_2_ given by Eqs. (19) and (20) through simulations with parameters values given in Table 2. The blue region in parameter space of (*ω*, ε) corresponds to the stability zone of the endemic equilibrium point.

### Expression of *R*_0_ as function of climatic variables

Some parameters in the *R*_0_ expression (Eq. 10) are assumed to be constant while other parameters are considered to be temperature and relative humidity sensitive over space and time. The actual expression of *R*_0_ (Eq. 10) is obtained with the following assumptions: (i) fungal lethal time is temperature sensitive; (ii) the proportion of fungal growth media (pest insects) allocated towards CFUs (mycelium/ conidia) is temperature and relative humidity sensitive; (iii) the parameters *α*, *β*, *K* and *γ* are constants. Temperature sensitive relation of fungal growth rate is described in^31^. Data obtained in^60^ were employed to estimate the parameters linking temperature, relative humidity and the proportion of resources (insects) available for spore production. Table 3 contains the summary of the parameters ε and *ω.*

### Mapping *R*_0_ under climate (relative humidity and temperature) variability

To map *R*_0_, its expression as function of climatic (relative humidity and temperature) variables was used. A matrix of geographical coordinates was built within the extent of the area of interest, afterward for each point coordinate of the matrix; we extracted the monthly average value of the temperatures and relative humidity from the respective raster layer and estimated the value of *R*_0_ using its mathematical expression (Eq. 10). The resulting matrix of *R*_0_ is converted into American Standard Code for Information Interchange (ASCII) files through spatial interpolation which was uploaded into Quantum-Geographical Information System (Q-GIS version 3.10)^84^ for visualization and mapping. Subsequently, the potential areas where locusts outbreak effects can be attenuated if the entomopathogenic fungus is introduced as a biocontrol agent are visualized. Overall, the approach exploits the spatial variation of temperature and relative humidity to provide predictions of the entomopathogenic fungus efficiency at large scale. The process was implemented in R (V.3.6.3)^85^ using several R-packages that include.

### Sensitivity analysis (SA), model evaluation and validation

A sensitivity analysis was performed to determine how sensitive the model output *R*_0_ (Eq. 10) is to changes in the model parameters, which further allows to determine which climate variable (relative humidity, temperature) that have most influence on the stability/instability of the equilibrium points and on *R*_0_. Sensitivity analysis provides a way to measure how changes in the parameters translate into variations in *R*_0_. It also assesses the relative importance of different factors responsible for the efficacy of the entomopathogenic fungus as a biocontrol agent against locusts and to better determine how to improve it. Sensitivity analysis was performed by the Partial Rank Correlation Coefficient (PRCC) algorithm^86^, where large PRCC values (> 0.5 or < − 0.5) indicate that the parameter has a high influence (positive/negative) on the model outcome. A positive PRCC value indicates the increase of the efficacy with the increase of the parameter and a negative value indicates the decrease of the efficacy with an increase of the parameter.

A recent study in some East African countries predicted the breeding area for desert locust under climate change scenario using temperature, rainfall and, sand and soil moisture content as key bioclimatic factors^68^. The maps obtained in^68^, that predicted the current and future area of suitability in Kenya was used to compare if, the locations predicted to be highly suitable for the long-term establishment of desert locust, fits with the areas predicted by the current model to be deemed adequate for applying the entomopathogenic fungus as a biocontrol agent, against locust at a specific month. Furthermore, to evaluate the ability of the spatial projection of *R*_0_ as a tool to predict the suitable month for applying the entomopathogenic fungus as a biocontrol agent at a given location, the point coordinates of the locust incidences in the respective countries were uploaded to the maps of *R*_0_ to compare and check how these points fit with the areas having *R*_0_ greater than one (*R*_0_ > 1).

## Data Availability

All data generated or analysed during this study are included in this published article.
